# The Massive Online Needs Assessment (MONA) to inform the development of an emergency haematology educational blog series

**DOI:** 10.1007/s40037-018-0406-0

**Published:** 2018-02-27

**Authors:** Teresa M. Chan, David Jo, Andrew W. Shih, Vinai C. Bhagirath, Lana A. Castellucci, Calvin Yeh, Brent Thoma, Eric K. Tseng, Kerstin de Wit

**Affiliations:** 1 Division of Emergency Medicine, Department of Medicine, McMaster University, Hamilton, Ontario Canada; 2 McMaster Education Research, Innovation, and Theory (MERIT), Hamilton, Ontario Canada; 3 Michael G. DeGroote School of Medicine, McMaster University, Hamilton, Ontario Canada; 4 Department of Pathology and Molecular Medicine, University of British Columbia, Vancouver, Canada; 5 Division of Hematology and Thromboembolism, Department of Medicine, McMaster University, Hamilton, Ontario Canada; 6 Division of Hematology, Department of Medicine, University of Ottawa, Ottawa, Canada; 7 Royal College of Physicians and Surgeons of Canada’s Emergency Medicine Training Program, University of Toronto, Toronto, Canada; 8 Department of Emergency Medicine, University of Saskatchewan, Saskatoon, Canada

**Keywords:** Needs assessment, Online educational resources, Free open access medical education (FOAM)

## Abstract

**Background:**

Online educational resources are criticized as being teacher-centred, failing to address learner’s needs. Needs assessments are an important precursor to inform curriculum development, but these are often overlooked or skipped by developers of online educational resources due to cumbersome measurement tools. Novel methods are required to identify perceived and unperceived learning needs to allow targeted development of learner-centred curricula.

**Objectives:**

To evaluate the feasibility of performing a novel technique dubbed the Massive Online Needs Assessment (MONA) for the purpose of emergency haematology online educational curricular planning, within an online learning community (affiliated with the Free Open Access Medical education movement).

**Methods:**

An online survey was launched on CanadiEM.org using an embedded Google Forms survey. Participants were recruited using the study website and a social media campaign (utilizing Twitter, Facebook, Blogs, and a poster) targeting a specific online community. Web analytics were used to monitor participation rates in addition to survey responses.

**Results:**

The survey was open from 20 September to 10 December 2016 and received 198 complete responses representing 6 medical specialties from 21 countries. Most survey respondents identified themselves as staff physicians (*n* = 109) and medical trainees (*n* = 75). We identified 17 high-priority perceived needs, 17 prompted needs, and 10 topics with unperceived needs through our MONA process.

**Conclusions:**

A MONA is a feasible, novel method for collecting data on perceived, prompted, and unperceived learning needs to inform an online emergency haematology educational blog. This methodology could be useful to the developers of other online education resources.

**Electronic supplementary material:**

The online version of this article (10.1007/s40037-018-0406-0) contains supplementary material, which is available to authorized users.

## Introduction

The Free Open Access Medical education (FOAM) movement encompasses the grassroots explosion of online medical educational and knowledge translation resources. FOAM seeks to minimize barriers to providing education and continued competency in medical care [1–3]. However, the FOAM movement has been criticized for being *ad hoc* in terms of generation of content. Critics cite an imbalance of resources focusing on trendy topics in emergency medicine and critical care [4]. Topics focussing on airway techniques, electrocardiography interpretation, resuscitation, and ultrasonography are overrepresented, while other topics such as haematological disorders (<0.6% of total content) are scarce [4]. Imbalanced representation of topics may lead to a disconnect between online curricula and clinical practice. Curriculum design principles commonly list needs assessment as a core planning procedure [5]. Yet, few FOAM producers engage in learner-centred strategies for discerning their audience’s needs.

CanadiEM (www.canadiem.org) is a multi-author educational emergency medicine blog, which provides free-access educational content for practising emergency physicians, residents, medical students, and other emergency healthcare providers. In 2016, CanadiEM was approached by a multidisciplinary team of haematology experts to host a practical, novel and accessible curriculum aimed at educating students, residents and physicians on emergency bleeding and clotting scenarios. We had noticed a lack of accessible and up-to-date material for practising clinicians, partly because of rapid developments in the clinical field. To inform the design of a learner-centred FOAM curriculum that addresses gaps in current curricula, we embarked on an online innovation that sought to engage our readership in a needs assessment [6, 7], we dubbed a Massive Online Needs Assessment (MONA). By sharing our process and findings, we hope that other FOAM producers may similarly engage their readership to identify priority educational content.

## Methods

### Setting

CanadiEM is a multi-author blog with a volunteer corps of approximately 50 editors who work collaboratively online to generate novel content for emergency providers. The site receives roughly a quarter of a million unique international site visits per year.

### Ethics

This study received approval from our institutional review board (HIREB: #2016-1954-GRA).

### Materials

Our needs assessment was created on Google Forms (Mountainview, CA, USA) and embedded on a CanadiEM.org blog post. The needs assessment included:

1. a section on the reader’s perceived needs;
2. a section which prompted them to identify needs (e. g. via story-telling around difficult cases); and then
3. a section with multiple-choice questions (designed to discern unperceived knowledge gaps).

The full survey can be found in the Online Electronic Supplementary Material, Appendix A & B. Our needs assessment was informed by previous literature, with the exception of the ‘prompted’ needs assessment part (i. e. difficult case description, Part 2) which is a novel innovation in our MONA survey [8].

### Participant recruitment

We recruited participants on social media via three promotional avenues, namely the CanadiEM blog, Twitter, and Facebook. Our 30-minute survey was available online from 20 September to 10 December 2016. Participants who completed the survey had an opportunity to enter a draw for gift cards.

### Outcomes

Topics endorsed by 30–50% of the participants were considered moderate priority while topics with >50% participant endorsement were considered high priority. The topics where <50% of participants answered questions correctly were identified as knowledge gaps. A misperception was defined as an incorrect response that >60% of participants selected.

### Analysis

Feasibility of our new MONA technique was defined *a priori *as 150 responses from at least 4 specialties in 4 or more countries. We felt this sample size was required to identify areas of knowledge gaps (defined *a priori* as questions where <50% of readers gave the correct responses). Participant demographics were analyzed with descriptive statistics using Microsoft Excel for Mac 2015 (Microsoft Corporation, Redmond, WA). A thematic analysis of data from the qualitative comments about possible topics and the difficult scenarios were analyzed by two investigators (TC, ET) and agreed upon by consensus with iterative rounds of discussion. Responses were coded by each author, in a constant comparison fashion.

## Results

During the study period, the CanadiEM webpage which hosted the link to the survey was visited 866 times by visitors at unique internet protocol addresses according to Google Analytics (Mountainview, CA, USA). A total of 198 participants from 6 specialty areas and 21 countries completed the MONA. This exceeded our feasibility goal of 150 participants, from at least 4 specialties and 4 countries. The participants comprised 109 physicians (55% respondents), 46 residents (23%), 29 medical students (15%), and other healthcare practitioners (*n* = 14). Of the respondents, 57% were identified as male (*n* = 113). The majority of respondents were from Canada (*n* = 115, 58%), and the United States (*n* = 51, 26%), with the remaining 32 respondents from a wide variety of 19 other countries. Most were Emergency Medicine providers (*n* = 118, 60%), with a strong showing from Internal Medicine (*n* = 41, 20%), and the remainder (*n* = 39) identifying with Primary Care, Surgical Specialties, Critical Care, Anaesthesia, or other groups.

### Part 1—Perceived needs results

Risk of thrombosis for reversal agents, adjunct treatments for acute bleeding, and reversal of anticoagulants were identified as topics of highest perceived need. We also identified 17 other high priority topics and 6 moderate priority topics. There were no additional topics via free-entry text responses.

### Part 2—Prompted needs results

Our thematic analysis revealed 17 unique topics. Most cases were focused on patients with a high bleeding risk and high risk of clotting (for example, acute venous thrombosis or mechanical heart valve). Interestingly, none of these topics were written within the optional free-text responses of Part 1.

### Part 3—Unperceived needs results

Knowledge gaps were identified in 10 of 15 questions. Most were associated with cases regarding perioperative reversal of anticoagulants, coagulopathic trauma patients, and deep vein thrombosis management. Only one question on the topic of pulmonary embolism diagnosis was identified as problematic, as participants correctly answered these questions with a high frequency. In 8 of 10 questions where a knowledge gap was identified, a misperception (one incorrect response selected >60% of the time) was responsible.

**Fig. 1:**
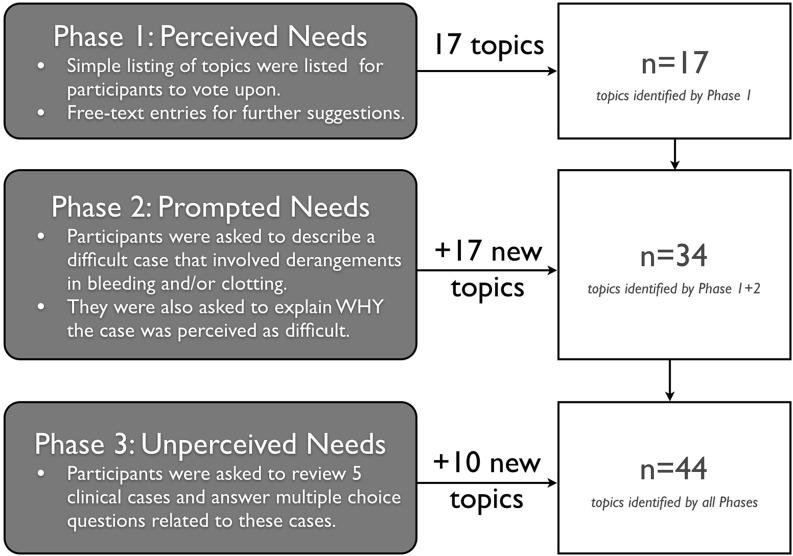
The contributions of each part of our Massive Online Needs Assessment (MONA)

Fig. 1 summarizes the contributions of each part of the needs assessment. Appendices C & D in the Online Electronic Supplementary Material detail our findings in full.

## Discussion

As shown previously with the METRIQ study [9, 10], it is possible to engage a learning community to assist with online surveys. We achieved our feasibility mark (i.e. >150 participants from more than 4 countries) in less than 4 months. Assuming each unique visit was from distinct individuals, the survey completion rate was 22% (198/866), which is similar to rates reported for other online surveys of physicians [11].

Asking participants to describe their needs through storytelling was a novel way to triangulate their learning needs (Part 2). We identified 17 additional areas of need, which the participants did not self-identify in Part 1 (i. e. when they could write in ‘other’ within the drop-down list). We feel that the most interesting lesson learned was that the ‘difficult case’ descriptions provided a unique window into the needs of our audience. In addition to the perceived needs, the descriptions also provided insight that more complex situations were important to address (e. g. when a patient who required antiplatelet drugs for a diagnosis such as new cardiac stents presented with a bleeding problem such as an intracranial haemorrhage). Furthermore, the multiple choice questions (Part 3) also helped to identify areas of unperceived needs, since this revealed where there was substantive variation and/or misperception on various topic areas.

When developing our curriculum, we anticipate that we will utilize Parts 1 and 3 to derive individual topics, and Part 2 results have led to the consideration of case-based discussions around complex scenarios and non-medical expert topics. This MONA has allowed us to gather useful information to create a new online curriculum.

## Limitations

We acknowledge that this has been a singular attempt at using a technique to establish the needs of an online learning community, and is restricted to a specific topic and specific online context. As this is a new tool, future studies will focus on the effect of MONA on educational outcomes. Further replication studies are required to validate MONA for other FOAM contexts and for other topics. We believe that multidisciplinary teams are not only key for content generation, but also for outreach. Two of the investigators (TC, BT) have been active within the online learning community [12], in part due to their involvement with CanadiEM, and this improved our ability to reach and/or influence members of this learning community to participate. Furthermore, we do not know whether the readers who chose not to complete the needs assessment are active online learners, or whether they differ in their needs from the people who completed the survey. This is a common problem for survey methodology and not unique to this MONA. We had 198 completed responses. Unfortunately, Google Forms does not record partially completed surveys and it is likely that data from potential participants were lost.

## Conclusion

Our MONA has demonstrated that it is possible to recruit members of an online learning community to assist educators to determine both perceived and unperceived learning needs in an emergency haematology blog. The data gathered from our MONA will help us create a targeted curriculum. Online educators may choose to use such a technique to better quantify and qualify the needs of their audiences.

## Caption Electronic Supplementary Material

Appendix A: Social Media for Knowledge Translation and Education 2 Needs Assessment Survey

Appendix B: Results of a case-based multiple-choice test created to identify unperceived needs

Appendix C: Topics in area of perceived needs within the domains of thrombosis and clotting, acute bleeding, and treatment and therapy of thrombosis and bleeding

Appendix D: Topics in areas of perceived needs from the difficult scenario descriptions
