# Supplementary material for: The Massive Online Needs Assessment (MONA) to inform the development of an emergency haematology educational blog series
Source: Perspect Med Educ. 2018 Feb 27;7(3):219–23. doi: 10.1007/s40037-018-0406-0 (PMC6002288; doi:10.1007/s40037-018-0406-0)
Supplement: Supplementary file 1 — Appendix A: Social Media for Knowledge Translation and Education 2 Needs Assessment Survey [file 40037_2018_406_MOESM1_ESM.pdf]

Social Media for Knowledge Translation and Education 2

You are invited to participate in a study titled Social Media for Knowledge Translation and Education 2 conducted by Dr. Teresa Chan, Department of Emergency Medicine, McMaster University.

Purpose and Objectives
The main purpose of this study is to assess the usefulness of an online needs assessment disseminated through social media in the generation of free open access medical education (FOAM) resources. Our study will use this online survey to evaluate the perceived and unperceived needs of the FOAM movement participants, specifically with regards to thrombosis and bleeding management. Experts in thrombosis and bleeding will be able to use the results to create appropriate and useful resources.

What is involved?
If you agree to voluntarily participate in this research, you will be asked to complete an online survey. The survey consists of a few demographic questions as well as both open ended and closed questions about thrombosis and bleeding to assess perceived and unperceived needs.

Compensation
All participants will be entered into a draw for one of four $250 gift cards to Amazon.com.

Confidentiality
Minimal identifiable information will be collected in our study. All collected information will be password protected and stored securely within the McMaster University network.

Potential Risks
There are no known or anticipated risks to you by participating in this research.

Right to Withdraw
Your participation is completely voluntary. You may withdraw from the research project at any time or for any reason, without explanation or penalty from study investigators.

Research Team
Dr. Teresa Chan, McMaster University, teresa.chan@medportal.ca
David Jo, McMaster University, david.jo@medportal.ca
Dr. Kerstin De Wit, McMaster University, dewitk@mcmaster.ca
Dr. Vinai Bhagirath, McMaster University, bhagiv@mcmaster.ca
Dr. Andrew Shih, McMaster University, andrew.shih@medportal.ca
Dr. Calvin Yeh, McMaster University, yehch@mcmaster.ca
Dr. Lana Castellucci, University of Ottawa, lcastellucci@toh.on.ca
Dr. Brent Thoma, University of Saskatchewan, brent.thoma@usask.ca

Questions or Concerns
If you have any questions or concerns please contact Dr. Teresa Chan, Department of Emergency Medicine, McMaster University, telephone: 905-521-2100 ext. 76993, or email: teresa.chan@medportal.ca. In addition, you may verify the ethical approval of this study, or raise any concerns you might have, by contacting the Hamilton Integrated Research Ethics Board, telephone: 905.521.2100.

* Required

Clicking “I agree” below indicates that you are giving consent to participate in this study, that you have read and understood the information provided, have had an opportunity to ask questions, and those questions have been answered to your satisfaction. *

- [ ] I agree

Continue »

11% completed

Powered by

This form was created inside of medportal.

Report Abuse - Terms of Service - Additional Terms

Social Media for Knowledge Translation and Education 2

* Required

Demographic Data

What is the best descriptor of your present profession? (If you are a trainee, please check the profession for which you are training to enter) *

- ◯ Physician
- ◯ Nurse
- ◯ Respiratory Therapist
- ◯ Pharmacist
- ◯ Paramedic
- ◯ Physician Assistant
- ◯ Nurse Practitioner
- ◯ Other: ____________

Do you have an area of specialty? *

- ◯ Internal Medicine
- ◯ Emergency Medicine
- ◯ Critical Care
- ◯ Primary Care (Family Medicine, General Practitioner, etc.)
- ◯ Anesthesiology
- ◯ Surgical Specialty
- ◯ Other: ____________

Year of university degree allocation (if applicable)

____

Type of practice setting (if applicable)

- ◯ Hospital, University-affiliated
- ◯ Hospital, community
- ◯ Outpatient Clinic

Number of years practicing (put 0 if still a trainee) *

Number of years in training if currently a trainee (e.g. PGY1, 2, F1, F2... etc)

Gender *

- Male
- Female
- Other

Country of practice *

Language of medical training *

« Back   Continue »

22% completed

Powered by

This form was created inside of medportal.

Report Abuse - Terms of Service - Additional Terms

Social Media for Knowledge Translation and Education 2

Perceived Needs

What topics would you like to learn about, with regards to thrombosis/clotting issues?

- [ ] Diagnosis of venous thrombosis
- [ ] Choice of anticoagulants for venous thrombosis
- [ ] Choice of anticoagulants for stroke prevention in atrial fibrillation
- [ ] Anticoagulation in cancer patients
- [ ] Anticoagulation with renal impairment
- [ ] Anticoagulation in pregnancy
- [ ] Interruption of anticoagulation for procedures
- [ ] Decisions on duration of anticoagulation for venous thrombosis
- [ ] Risk of thrombosis for reversal agents

What topics would you like to learn about with regards to issues around acute bleeding?

- [ ] Risk scores for bleeding
- [ ] Monitoring new anticoagulants
- [ ] Massive transfusion protocols
- [ ] Adjunct treatments for acute bleeding
- [ ] Acute management of inherited bleeding disorders
- [ ] Interpretation of DIC workup, iron deficiency bloodwork, and erthryopoietin levels levels
- [ ] Management of the acutely bleeding pregnant woman/woman of childbearing age
- [ ] Managing acute venous thrombosis in the bleeding patient

What topics would you like to learn about with regards to treatments and therapy?

- [ ] Reversal of anticoagulants
- [ ] Treatment of coagulopathies
- [ ] Appropriateness of transfusion (indications for transfusion, choice of blood products, hemovigilance)
- [ ] Adverse Effects of Transfusion including best practices for diagnosis and reporting
- [ ] Periprocedural triggers for Transfusion
- [ ] Choice of anticoagulants for venous thrombosis
- [ ] Choice of anticoagulants for stroke prevention in atrial fibrillation

« Back Continue »

33% completed

Powered by

This form was created inside of medportal.

Report Abuse - Terms of Service - Additional Terms

Social Media for Knowledge Translation and Education 2

Unperceived Needs

Please describe a difficult clinical scenario you had due to clotting or bleeding, or where you had a patient with a blood disorder. Please do not put confidential information such as name or location. Keep your story generic.

Please tell us WHY the scenario was difficult for you?

Onward to the Scenarios

The next phase of this will be 5 scenarios with some associated multiple choice questions. Please answer these questions to the best of your ability.

« Back Continue »

44% completed

Powered by

This form was created inside of medportal.

Report Abuse - Terms of Service - Additional Terms

Social Media for Knowledge Translation and Education 2

Scenario 1

Maria is a 27-year-old female university student with no past medical or surgical history, and has been taking an oral contraceptive pill for the past 8 years. She has never smoked. 2 days ago, she developed a new sharp pain on her right upper back. Initially she thought this to be a muscle strain from power yoga, but it has been getting progressively worse and is painful when she breathes in. She denies fever or chills, but says that she seems to be having a dry cough when exerting for her daily cycling class and has felt tired during classes. She denies shortness of breath or syncope. She tells you, “I know it’s probably just the flu now, but I want to be sure, because my grandmother had a blood clot”.

On examination, Maria is a well-appearing, average sized young woman. Her vitals reveal T=37C, HR 85, BP 110/90, RR 15, SpO2 99% RA. Head and neck exam is normal. Cardiorespiratory examination is normal and there is no jugular venous distension. The chest pain is not reproducible with palpation or movement. She does endorse mild bilateral calf pain that she attributes to running. ECG performed at triage shows NSR and no signs of S1Q3T3 or RBBB.

What is the pre-test probability for pulmonary embolism?

- ◯ Unlikely: Given her age, she probably has a viral infection and/or musculoskeletal chest pain
- ◯ Unlikely: Her Wells Score is <4.5; does not have signs or symptoms of DVT or PE, and has no past medical or surgical history
- ◯ Likely: She has a new exertional cough without signs and symptoms of infection, and has been on a long term oral contraceptive.
- ◯ Likely: There is no alternative diagnosis more likely than PE
- ◯ Insufficient information

What steps should be taken next in investigation?

- ◯ This is highly unlikely to be a PE. Discharge home with instructions to monitor for change or worsening of pain and/or shortness of breath
- ◯ This has a low likelihood of being a PE because she is a long-time oral contraceptive user. D-dimer may be employed to rule out VTE.
- ◯ This has a moderate likelihood of being a PE because of the acute and worsening symptoms, cough with exertion, and long-term exposure to exogenous hormones. She may be given a dose of LMWH or a DOAC with instructions to return for CTPA in the morning.
- ◯ This has a moderate likelihood of being a PE because of the acute and worsening symptoms, cough with exertion, and long-term exposure to exogenous hormones. She may be given a dose of LMWH or a DOAC with instructions to return for V/Q scan in the morning.
- ◯ This has a moderate likelihood of being a PE because of the acute and worsening symptoms, cough with exertion, and long-term exposure to exogenous hormones. She may be given a dose of LMWH or a DOAC and admitted for overnight observation and V/Q scan in the morning.

You discover that she has a family history of a hypercoagulable disorder. How does this change management?

- This is highly unlikely to be a PE. Discharge home with instructions to monitor for change or worsening of pain and/or shortness of breath. Thrombosis service may be consulted.
- This has a low likelihood of being a PE. D-dimer may be employed to rule out VTE. Thrombosis service may be consulted.
- She may be given a dose of LMWH or a DOAC with instructions to return for CTPA in the morning.
- She may be given a dose of LMWH or a DOAC with instructions to return for V/Q scan in the morning.
- She may be given a dose of LMWH or a DOAC and admitted for overnight observation and V/Q scan in the morning.

« Back Continue »

55% completed

Powered by

This form was created inside of medportal.

Report Abuse - Terms of Service - Additional Terms

Social Media for Knowledge Translation and Education 2

Scenario 2

A 68 year old man with history of atrial fibrillation taking warfarin for stroke prevention is preparing for elective hip arthroplasty. His co-morbidities are hypertension, type II diabetes, and overweight. There is no history of bleeding or thromboembolism. One week pre-operatively, blood work reveals: hemoglobin 142g/L, platelets 180 x109/L, creatinine 75μM, and INR 2.3.

How should you manage this patient's antithrombotics pre-operatively?

- ◯ Stop warfarin with last dose 3 days before the scheduled surgery, with no use of low molecular weight heparin pre-operatively.
- ◯ Stop warfarin with last dose 6 days before the scheduled surgery, with therapeutic doses of low molecular weight heparin from 3 days before the surgery until 24h before the surgery.
- ◯ Stop warfarin with last dose 6 days before the scheduled surgery, and start ASA 81mg daily at the same time.
- ◯ Continue warfarin through the procedure.
- ◯ Stop warfarin with last dose 6 days before the scheduled surgery, with no use of low molecular weight heparin pre-operatively.

You elect to stop the warfarin with no use of low molecular weight heparin pre-operatively. The patient undergoes the hip arthroplasty with estimated blood loss of 300 mL. There is no ongoing bleeding 8h after the surgery. The hemoglobin post-operatively is 118g/L, creatinine 67μM, and all other values normal. How would you manage the patient's antithrombotic medications?

- ◯ Resume warfarin 5 days post-operatively to ensure no further bleeding, with no use of other anticoagulants post-operatively.
- ◯ Resume warfarin the evening after the surgery with no use of other anticoagulants post-operatively.
- ◯ Resume warfarin the evening after the surgery, and use low molecular weight heparin at therapeutic doses from 48h post-operatively until a therapeutic INR is attained.
- ◯ Resume warfarin the evening after the surgery, and use a direct-acting anticoagulant or low molecular weight heparin at prophylactic doses for venous thromboembolism prevention until a therapeutic INR is attained.
- ◯ Use a direct-acting anticoagulant or low molecular weight heparin at prophylactic doses for venous thromboembolism prevention for 30-35 days and then resume warfarin.

What recent large randomized clinical trial is directly relevant to the perioperative management of this patient's anticoagulation (study acronym, first author, or DOI)?

« Back

Continue »

66% completed

Powered by

This form was created inside of medportal.

Report Abuse - Terms of Service - Additional Terms

Social Media for Knowledge Translation and Education 2

Scenario 3

You are seeing a 42-year old man in the ER who is post-MVC and is brought into the trauma bay. He was bleeding at the scene and was resuscitated en route to the trauma bay. Vitals in the trauma bay are 106/69 mmHg, HR 121, 96% RA, and afebrile. His initial CBC shows that his hemoglobin is 72 g/L (7.2 g/dL, 4.47 mmol/L, Hct 22%), platelet count of 186 x 109/L (186 x 103/mcL), and a WBC of 8.7 x 106/L (8.7 x 103/mcL). An initial coagulation screen demonstrates an INR of 3.3 and a PTT within reference lab ranges. On his Medic-Alert bracelet, he is found to be on warfarin. He has no known drug allergies according to the medical chart. He is rousable but confused.

How would you reverse his warfarin to try and stop the bleeding? (check all that apply)

- ☐ Frozen Plasma
- ☐ Prothrombin Complex Concentrates
- ☐ Solvent Detergent Plasma (reduced infectious risk compared to Frozen Plasma)
- ☐ Fibrinogen Concentrates
- ☐ Vitamin K 10mg IV
- ☐ Tranexamic Acid

For his symptomatic anemia, what red blood cells would you transfuse (if any)?

- ○ Crossmatch-compatible O negative
- ○ Least-incompatible O negative
- ○ Uncrossmatched O positive
- ○ Crossmatch-compatible O positive
- ○ Given his hemoglobin is not <70 g/L, do not transfuse

A massive hemorrhage protocol is initiated and the patient is taken to the OR for his traumatic injuries. The following must be considered (check all that apply).

- ○ Calcium and magnesium monitoring
- ○ Blood group and screen
- ○ Acute normovolemic hemodilution to reduce transfusion burden
- ○ Repeat INR before surgery after PCC administration within 2 hours post-administration
- ○ Blood warmer to prevent hypothermia

« Back   Continue »

77% completed

Powered by

This form was created inside of medportal.

Report Abuse - Terms of Service - Additional Terms

Social Media for Knowledge Translation and Education 2

Scenario 4

You are evaluating a 75 year old man on the medical ward, to organize his discharge home. You notice that his ECG shows atrial fibrillation, rate 88 beats/min. He is well, his BP is 134/76. He has a history of hypertension and colon cancer (in remission). He is prescribed atorvastatin, amlodipine, metoprolol and aspirin.

What is his yearly risk of stroke?

- 0.5%
- 4%
- 1%
- 6%
- 9%

In order to choose an anticoagulant, what is the most important information to obtain?

- How long has he taken aspirin?
- Has he had any falls at home?
- Serum creatinine
- His hemoglobin
- His liver function

You have started rivaroxaban for this man. You should

- Stop his aspirin immediately
- Stop his aspirin in 2 days time
- Continue his aspirin
- Stop his aspirin in a week
- Leave it up to the patient to decide whether to continue the aspirin

« Back Continue »

88% completed

Powered by

This form was created inside of medportal.

Report Abuse - Terms of Service - Additional Terms

Social Media for Knowledge Translation and Education 2

Scenario 5

71F presents with a several week history of right leg swelling. This has been progressive and persistent swelling. She denies symptoms of chest pain, pleurisy, and shortness of breath. Her PMHx includes hypothyroidism, hypertension, GERD, and depression. Her medications include el-troxin, bisoprolol, pantoprazole, Effexor. Vitals are stable and examination of the right leg reveals pitting edema to the mid-shin and tenderness to palpation of the calf. An ultrasound confirms diagnosis of deep vein thrombosis (DVT) involving the popliteal vein and femoral vein of the right leg. Her lab parameters are: Hb 107 g/L, Plt 179 x109, creatinine clearance (CrCl) 56 mL/min (calculated by Cockcroft-Gault). Additional history reveals no provoking factors such as trauma, immobilization or recent surgery. There is no history of malignancy and no previous bleeding concern. She reports 30 lb weight loss over the last 6 months.

What treatment options are available for this patient?

- ◯ Low-molecular-weight-heparin (LMWH) in combination with vitamin K antagonist (VKA) with target INR of 2.0-3.0
- ◯ IV unfractionated heparin (UFH) in combination with vitamin K antagonist (VKA) with target INR of 2.0-3.0
- ◯ Rivaroxaban 15 mg BID X 3 weeks then 20 mg daily
- ◯ Apixaban 10 mg BID X 7 days then 5 mg BID
- ◯ LMWH for 5 days then dabigatran 150 mg twice daily

Due to the unprovoked nature of the DVT you want to perform malignancy screening on your patient. What tests should you consider?

- ◯ Complete history and physical examination, routine investigations: CBC, electrolytes, renal function, LFTs, CXR
- ◯ Upper and lower endoscopy
- ◯ Mammography/pap smear
- ◯ CT scan of the abdomen
- ◯ Fecal occult blood

Due to the presence of anemia in your post-menopausal patient, a colonoscopy has been arranged. She is anticoagulated with rivaroxaban 20 mg daily. How to you manage her anticoagulation around the time of procedure?

- ◯ Continue anticoagulant
- ◯ Measure INR the day before surgery and base decision on the result
- ◯ Hold rivaroxaban 5 days before the procedure and given low molecular weight heparin bridging

- Hold rivaroxaban starting on pre-op day 1
- Hold rivaroxaban starting on pre-op day 2

« Back Submit

100%: You made it.

Never submit passwords through Google Forms.

Powered by

This form was created inside of medportal.

Report Abuse - Terms of Service - Additional Terms
