# Supplementary material for: The Massive Online Needs Assessment (MONA) to inform the development of an emergency haematology educational blog series
Source: Perspect Med Educ. 2018 Feb 27;7(3):219–23. doi: 10.1007/s40037-018-0406-0 (PMC6002288; doi:10.1007/s40037-018-0406-0)
Supplement: Supplementary file 2 — Appendix B: Results of a case-based multiple-choice test created to identify unperceived needs [file 40037_2018_406_MOESM2_ESM.pdf]

Appendix B. Results of a case-based multiple-choice test created to identify unperceived needs. Knowledge gaps were defined as less than 50% of participants correctly answering a question. Misperceptions were defined as greater than 60% of participants choosing the same incorrect answer.

Case	Question	Correct n (%)	Knowledge gap	Misperception
**Case 1**<br>Maria is a 27-year-old female university student with no past medical or surgical history, and has been taking an oral contraceptive pill for the past 8 years. She has never smoked. 2 days ago, she developed a new sharp pain on her right upper back. Initially she thought this to be a muscle strain from power yoga, but it has been getting progressively worse and is painful when she breathes in. She denies fever or chills, but says that she seems to be having a dry cough when exerting for her daily cycling class and has felt tired during classes. She denies shortness of breath or syncope. She tells you, "I know it's probably just the flu now, but I want to be sure, because my grandmother had a blood clot".<br><br>On examination, Maria is a well-appearing, average sized young woman. Her vitals reveal T=37C, HR 85, BP 110/90, RR 15, SpO2 99% RA. Head and neck exam is normal. Cardiorespiratory examination is normal and there is no jugular venous distension. The chest pain is not reproducible with palpation or movement. She does endorse mild bilateral calf pain that she attributes to running. ECG performed at triage shows NSR and no signs of S1Q3T3 or RBBB.	1A. What is the pre-test probability for pulmonary embolism?	101 (51.0)	No	No
	1B. What steps should be taken next in investigation?	139 (70.2)	No	No
	1C. You discover that she has a family history of a hypercoagulable disorder. How does this change management?	95 (48.0)	**Yes**	No
**Case 2**<br>A 68 year old man with history of atrial fibrillation taking warfarin for stroke prevention is preparing for elective hip arthroplasty. His co-morbidities are hypertension, type II diabetes, and overweight. There is no history of bleeding or thromboembolism. One week pre-operatively, blood work reveals: hemoglobin 142g/L, platelets 180 x10^9/L, creatinine 75µM, and INR 2.3.	2A. How should you manage this patient's antithrombotics pre-operatively?	42 (21.2)	**Yes**	**Yes**
	2B. You elect to stop the warfarin with no use of low molecular weight heparin pre-operatively. The patient undergoes the hip arthroplasty with estimated blood loss of 300 mL. There is no ongoing bleeding 8h after the surgery. The hemoglobin post-operatively is 118g/L, creatinine 67µM, and all other values normal. How would you manage the patient's antithrombotic medications?	67 (33.8)	**Yes**	**Yes**
	2.3 What recent large randomized clinical trial is directly relevant to the perioperative management of this patient's anticoagulation (study acronym, first author, or DOI)?	47 (23.7)	**Yes**	**Yes**

**Case 3**<br>You are seeing a 42-year old man in the ER who is post-MVC and is brought into the trauma bay. He was bleeding at the scene and was resuscitated en route to the trauma bay. Vitals in the trauma bay are 106/69 mmHg, HR 121, 96% RA, and afebrile. His initial CBC shows that his hemoglobin is 72 g/L (7.2 g/dL, 4.47 mmol/L, Hct 22%), platelet count of 186 x 109/L (186 x 103/mcL), and a WBC of 8.7 x 106/L (8.7 x 103/mcL). An initial coagulation screen demonstrates an INR of 3.3 and a PTT within reference lab ranges. On his Medic-Alert bracelet, he is found to be on warfarin. He has no known drug allergies according to the medical chart. He is rousable but confused.	How would you reverse his warfarin to try and stop the bleeding? (check all that apply)	42 (21.2)	**Yes**	**Yes**
	For his symptomatic anemia, what red blood cells would you transfuse (if any)?	70 (35.4)	**Yes**	**Yes**
	A massive hemorrhage protocol is initiated and the patient is taken to the OR for his traumatic injuries. The following must be considered (check all that apply).	43 (21.7)	**Yes**	**Yes**
**Case 4**<br>You are evaluating a 75 year old man on the medical ward, to organize his discharge home. You notice that his ECG shows atrial fibrillation, rate 88 beats/min. He is well, his BP is 134/76. He has a history of hypertension and colon cancer (in remission). He is prescribed atorvastatin, amlodipine, metoprolol and aspirin.	What is his yearly risk of stroke?	126 (63.6)	No	No
	In order to choose an anticoagulant, what is the most important information to obtain?	101 (51.0)	No	No
	You have started rivaroxaban for this man. You should (choose one)	128 (64.6)	No	No
**Case 5**<br>71F presents with a several week history of right leg swelling. This has been progressive and persistent swelling. She denies symptoms of chest pain, pleurisy, and shortness of breath. Her PMHx includes hypothyroidism, hypertension, GERD, and depression. Her medications include eltroxin, bisoprolol, pantoprazole, Effexor. Vitals are stable and examination of the right leg reveals pitting edema to the mid-shin and tenderness to palpation of the calf. An ultrasound confirms diagnosis of deep vein thrombosis (DVT) involving the popliteal vein and femoral vein of the right leg. Her lab parameters are: Hb 107 g/L, Plt 179 x109, creatinine clearance (CrCl) 56 mL/min (calculated by Cockcroft-Gault). Additional history reveals no provoking factors such as trauma, immobilization or recent surgery. There is no history of malignancy and no previous bleeding concern. She reports 30 lbs weight loss over the last 6 months.	What treatment options are available for this patient? (check all that apply)	7 (3.53)	**Yes**	**Yes**
	Due to the unprovoked nature of the DVT you want to perform malignancy screening on your patient. What tests should you consider? (check all that apply)	14 (7.07)	**Yes**	**Yes**
	Due to the presence of anemia in your post-menopausal patient, a colonoscopy has been arranged. She is anticoagulated with rivaroxaban 20 mg daily. How to you manage her anticoagulation around the time of procedure?	54 (27.3)	**Yes**	No
