# Supplementary material for: The Massive Online Needs Assessment (MONA) to inform the development of an emergency haematology educational blog series
Source: Perspect Med Educ. 2018 Feb 27;7(3):219–23. doi: 10.1007/s40037-018-0406-0 (PMC6002288; doi:10.1007/s40037-018-0406-0)
Supplement: Supplementary file 3 — Appendix C: Topics in area of perceived needs within the domains of thrombosis and clotting, acute bleeding, and treatment and therapy of thrombosis and bleeding [file 40037_2018_406_MOESM3_ESM.pdf]

Appendix C. Topics in area of perceived needs within the domains of thrombosis and clotting, acute bleeding, and treatment and therapy of thrombosis and bleeding.

Topic	Number of Survey Respondents (%)
**Thrombosis and clotting**	
Risk of thrombosis for reversal agents	142 (71.7)
Interruption of anticoagulation for procedures	133 (67.2)
Anticoagulation in pregnancy	113 (57.1)
Anticoagulation with renal impairment	111 (56.1)
Decisions on duration of anticoagulation for venous thrombosis	110 (55.6)
Anticoagulation in cancer patients	76 (38.4)
Diagnosis of venous thrombosis	66 (33.3)
**Acute bleeding**	
Adjunct treatments for acute bleeding	132 (66.7)
Managing acute venous thrombosis in the bleeding patient	125 (63.1)
Monitoring new anticoagulants	124 (62.6)
Massive transfusion protocols	117 (59.1)
Interpretation of DIC workup, iron deficiency blood work, and erythropoietin levels	113 (57.1)
Management of the acutely bleeding pregnant woman/woman of childbearing age	109 (55.1)
Risk scores for bleeding	95 (48.0)
Acute management of inherited bleeding disorders (including hemophilia)	95 (48.0)
**Treatment and therapy of thrombosis and bleeding**	
Reversal of anticoagulants	153 (77.3)
Treatment of coagulopathies	127 (64.1)
Appropriateness of transfusion (indications for transfusion, choice of blood products, hemovigilance)	105 (53.0)
Adverse Effects of Transfusion including best practices for diagnosis and reporting	92 (46.5)
Peri-procedural triggers for Transfusion	67 (33.8)
Choice of anticoagulants for venous thrombosis	129 (65.2)
Choice of anticoagulants for stroke prevention in atrial fibrillation	104 (52.5)
