# Supplementary material for: The Massive Online Needs Assessment (MONA) to inform the development of an emergency haematology educational blog series
Source: Perspect Med Educ. 2018 Feb 27;7(3):219–23. doi: 10.1007/s40037-018-0406-0 (PMC6002288; doi:10.1007/s40037-018-0406-0)
Supplement: Supplementary file 4 — Appendix D: Topics in areas of perceived needs from the difficult scenario descriptions [file 40037_2018_406_MOESM4_ESM.pdf]

Appendix D Topics in areas of perceived needs from the difficult scenario descriptions.

<table>
<tr><th>Rank</th><th>Topic</th><th>Frequency</th><th>% of Cases</th><th>New Topic*</th></tr>
<tr><td>1</td><td>Weighing risks and benefits of bleeding & clotting
<table>
<tr><th>Sub-themes for Topic 1</th><th>Frequency</th></tr>
<tr><td>GI bleed with need for anticoagulation</td><td>17</td></tr>
<tr><td>ICH with need for anticoagulation</td><td>12</td></tr>
<tr><td>ACS with concurrent bleeding</td><td>6</td></tr>
<tr><td>Epistaxis in difficult situations (e.g. mechanical valve)</td><td>2</td></tr>
<tr><td>Other (e.g. Trauma)</td><td>5</td></tr>
</table></td><td>42</td><td>35.9%</td><td>Yes</td></tr>
<tr><td>2</td><td>VTE Management - Assessing need for anticoagulation (Starting new or restarting, or when to stop)
<table>
<tr><th>Sub-themes for Topic 2</th><th>Frequency</th></tr>
<tr><td>Restarting (e.g. anticoagulation held recently, when to restart)</td><td>3</td></tr>
<tr><td>Stopping (e.g. needs anticoagulation for prevention, but has new bleed)</td><td>3</td></tr>
<tr><td>Starting new (e.g. Afib, Subsegmental PE, Upper limb, DVT, Cancer-related DVT)</td><td>3</td></tr>
<tr><td>Anticoagulant Failure</td><td>1</td></tr>
<tr><td>Cerebral Venous Sinus Thrombosis</td><td>1</td></tr>
</table></td><td>11</td><td>9.4%</td><td>No</td></tr>
<tr><td>3</td><td>Reversal agents (including decision to reverse; how to handle antiplatelet agents)</td><td>8</td><td>6.8%</td><td>No</td></tr>
<tr><td>4</td><td>Coagulopathy of Liver Failure</td><td>7</td><td>6.0%</td><td>Yes</td></tr>
<tr><td>5</td><td>Periprocedural Anticoagulation / Coagulation Mgt (LP, Abdominal Surgery, Spinal anesthesia, Hemarthrosis)</td><td>6</td><td>5.1%</td><td>No</td></tr>
<tr><td>6</td><td>Choosing/Identifying Anticoagulation (DOACs, Coumadin, LMWH, others) vs. Antiplatelet (e.g. Prasugrel)</td><td>6</td><td>5.1%</td><td>No</td></tr>
<tr><td>7</td><td>Maintaining perfusion in bleeding patient (e.g. Massive Transfusion fpr Resuscitation)</td><td>5</td><td>4.3%</td><td>Yes</td></tr>
<tr><td>8</td><td>Inherited Bleeding Disorders (Hemophilia, Glanzmann thrombasthenia)</td><td>5</td><td>4.3%</td><td>Yes</td></tr>
<tr><td>9</td><td>DIC</td><td>4</td><td>3.4%</td><td>Yes</td></tr>
<tr><td>10 (tie)</td><td>Platelet transfusion & Hemostasis (e.g. ITP, bone marrow suppression, dialysis)</td><td>3</td><td>2.6%</td><td>Yes</td></tr>
<tr><td>10 (tie)</td><td>Gynecologic sources of bleeding</td><td>3</td><td>2.6%</td><td>Yes</td></tr>
<tr><td rowspan="12">Other bleeding and thrombosis topics identified</td><td>Pregnancy & VTE</td><td>2</td><td></td><td>No</td></tr>
<tr><td>Bleeding NYD</td><td>2</td><td></td><td>Yes</td></tr>
<tr><td>HIT</td><td>2</td><td></td><td>Yes</td></tr>
<tr><td>Alternatives to transfusion</td><td>1</td><td></td><td>Yes</td></tr>
<tr><td>Bleeding in Cancer/BM suppressed pt</td><td>1</td><td></td><td></td></tr>
<tr><td>DOACs & Thrombolysis in stroke</td><td>1</td><td></td><td>Yes</td></tr>
<tr><td>When to suspect lab error</td><td>1</td><td></td><td>Yes</td></tr>
<tr><td>Diagnosis of DVT</td><td>1</td><td></td><td>No</td></tr>
<tr><td>Cancer related DVT mgt</td><td>1</td><td></td><td>No</td></tr>
<tr><td>Sickle cell pts & transfusion</td><td>1</td><td></td><td>Yes</td></tr>
<tr><td>Coagulopathy of Trauma</td><td>1</td><td></td><td>Yes</td></tr>
<tr><td>Cost of medications</td><td>1</td><td></td><td>Yes</td></tr>
<tr><td colspan="5">* New topic identified that was not identified in any other phase of the needs assessment (e.g. survey free text, by investigators)</td></tr>
</table>
